# Development and Performance Analysis of a Semi-Supervised Gait Recognition Model for Pediatric Abnormalities Using a Hybrid Dataset

**DOI:** 10.3390/bioengineering13030272

**Published:** 2026-02-26

**Authors:** Xiaoneng Song, Kun Qian, Sida Tang

**Affiliations:** 1 Department of Physical Education, Jiangnan University, Wuxi 214122, China; 2 School of Artificial Intelligence and Computer Science, Jiangnan University, Wuxi 214122, China; 3 School of Electronic and Information Engineering, Wuxi University, Wuxi 214105, China

**Keywords:** gait recognition, Mean Teacher training strategy, spatial hierarchical pooling module

## Abstract

Pediatric gait abnormalities are closely intertwined with musculoskeletal dysfunctions and heightened injury risk, underscoring the urgency of early and accessible screening tools. Here, we develop and validate a video-based semi-supervised Abnormal Gait Recognition Module (AGRM) to address unmet needs in pediatric gait assessment, with a focus on diagnostic performance and clinical interpretability. The AGRM is built on a 3D ResNet backbone, synergistically integrated with a Mean Teacher Module (MTM) to mitigate the limitations of limited labeled clinical data, and a Spatial Hierarchical Pooling Module (SHPM) for robust multiscale spatiotemporal feature extraction—two core innovations tailored to gait dynamics. We trained and validated the model on a hybrid dataset combining self-collected pediatric gait videos and the public CASIA-B dataset, evaluating its performance in binary (normal vs. abnormal) and three-class (normal, genu varum, genu valgum) classification tasks using accuracy, macro-precision, macro-recall, and macro-F1 score. Ablation studies quantified the incremental contributions of MTM and SHPM, while Grad-CAM visualization was employed to enhance model interpretability. In the three-class classification task, the AGRM achieved a 70.5% accuracy, 72.1% macro-precision, 71.5% macro-recall, and a macro-F1 score of 0.718; in the binary task, it yielded a 80.3% precision and 79.2% recall. SHPM significantly augmented spatiotemporal feature aggregation, capturing fine-grained gait dynamics, whereas MTM improved model generalization under constrained labeled data scenarios—findings corroborated by ablation experiments. Grad-CAM visualization confirmed the model’s targeted attention to lower extremity regions, particularly the knee joints, aligning with the pathological loci of gait abnormalities. Collectively, our AGRM demonstrates robust performance and generalization in identifying pediatric gait abnormalities, while effectively capturing key pathological gait characteristics. This video-based intelligent approach offers a promising tool for early gait screening in both clinical and community settings, addressing barriers to accessible pediatric musculoskeletal assessment.

## 1. Introduction

Advances in data acquisition and computational capabilities have accelerated the integration of machine learning and probabilistic modeling into complex biomechanical analysis. Within this domain, gait recognition has become a practical approach for assessing postural health and supporting screening and triage by identifying gait patterns that may warrant further orthopedic/pediatric evaluation, rather than providing clinical diagnosis [1]. Abnormal gait not only compromises postural stability but also increases mechanical load and the risk of injury throughout the musculoskeletal system. Schroth [2] reported that 70.3% of patients with scoliosis have a knee or foot valgus, highlighting a strong association between spinal curvature, lower extremity malalignment, and gait irregularities. Such findings emphasize the potential of gait analysis for early intervention and the prevention of secondary disorders.

Lower limb malalignments, most notably genu valgum and genu varum, alter the distribution of load across the femur and modify the contact moments of the knee joint during locomotion, accelerating asymmetric cartilage wear and increasing the likelihood of osteoarthritis [3,4,5]. For example, Shetty et al. [6,7] reported genu varum in 40% of adult males and 28% of adult females in the Philippines, while Muller [8] stressed that corrective interventions during the critical period of gait development (ages 5 to 6) are essential to prevent long-term musculoskeletal dysfunction. Consequently, gait recognition in pediatric populations serves not only as a diagnostic and rehabilitative tool but also as an objective foundation for postural health promotion.

Contemporary gait research predominantly addresses identity recognition, disease diagnosis, and rehabilitation evaluation [9,10,11]. However, conventional recognition techniques often struggle with large-scale, high-dimensional datasets and the preservation of full spatiotemporal correlations, leading to feature degradation and suboptimal classification accuracy [12,13,14]. Common deep learning architectures such as R(2 + 1)D, UniFormer, C3D, and 3D ResNet have demonstrated promise in supervised settings [15,16,17] but face persistent limitations when applied to unlabeled data. These limitations include: (1) Restricted learning from unlabeled data without sufficient supervisory signals: The models tend to overfit to noise or irrelevant features, reducing temporal pattern discrimination. (2) The limited sample diversity, due to insufficient intra-class and inter-class variability, hampers the learning of generalizable features. (3) Domain shift discrepancies between the source and target data distributions impair transferability. (4) Class imbalance: Infrequent classes in unlabeled datasets exacerbate the risk of misclassification. (5) The Lack of task-specific guidance: Generic representations obtained in unsupervised contexts may not align with task-relevant features.

To address these challenges, recent research has adopted semi-supervised frameworks, such as the Mean Teacher approach, which have proven effective in leveraging unlabeled data to enhance model robustness [18,19,20]. In this paradigm, a student model is trained along with a teacher model whose parameters are updated through an Exponential Moving Average (EMA) of the student’s weights. The incorporation of consistency regularization ensures that the model learns stable, noise-tolerant representations, thereby improving generalization in data-scarce scenarios.

In addition to this, multiscale feature extraction strategies, including maximum grouping and average grouping within multiscale motion aggregation frameworks, facilitate the capture of fine-grained discriminative details and broader structural patterns in gait sequences [21,22,23]. This integration improves resilience to covariates such as clothing variation, occlusion, and load transport.

The proposed the Abnormal Gait Recognition Model (AGRM) is intended as a low-cost, video-based screening support tool for the early identification of potential leg alignment-related gait abnormalities in community or school settings. It aims to assist triage by flagging cases that may warrant further clinical assessment. AGRM is not intended to provide clinical diagnosis, severity grading, or treatment decisions. Individuals with pronounced or progressive abnormalities should be referred to orthopedic/pediatric specialists for standard clinical evaluation.

In this study, we introduce AGRM, which integrates a Mean Teacher semi-supervised learning strategy with a Spatial Hierarchical Pooling Module (SHPM). This design maximizes the utility of both labeled and unlabeled gait data, while improving sensitivity to multilevel structural changes in gait. The primary contributions of this work are as follows: (1) A semi-supervised AGRM framework employing a 3D ResNet backbone with Mean Teacher integration effectively exploits unlabeled gait datasets. (2) The Spatial Hierarchical Pooling Module captures hierarchical spatiotemporal features to improve the accuracy of abnormal gait classification. (3) Construction of hybrid datasets: We combine public datasets with a self-developed pediatric gait dataset and evaluate model performance on both binary and three-class classification tasks to validate accuracy and generalization between domains.

## 2. Methods

### 2.1. Abnormal Gait Recognition Model

**Figure 1:**
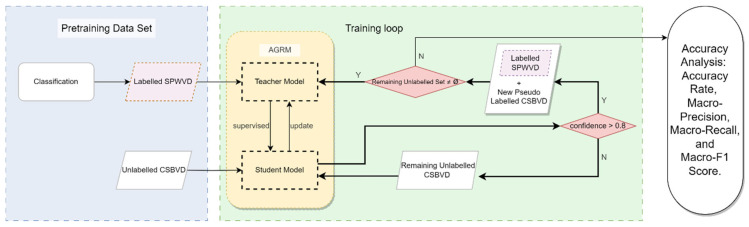
Flowchart of label processing.

Our proposed model builds upon a 3D ResNet architecture augmented with fully connected layers and integrates the MTM training strategy. Inside the 3D ResNet structure, we update the usage of max pooling to the SHPM. The integrated model is used to handle and process the data, producing video tensors, labels, and modified video tensors with added noise. The video tensor is fed into the student model, while the noisy video tensor is passed to the teacher model. Consistency loss is calculated through label consistency training, which updates the model weights. The feature classification is performed using SHPM, completing the model’s learning process. Using label consistency during the training phase and incorporating a Spatial Hierarchical Pooling Module for comprehensive feature extraction, the model effectively leverages information from both labeled and unlabeled data. If the calculated confidence rate, which comes from the prediction from both teacher and student models based on the same input, is higher than some specific value, then we consider it as an efficient pseudo-label and move this data input, together with the labeled dataset, as the new supervising dataset for the teacher model. When all unlabeled data get their added label, the process is finished. The detailed architecture of AGRM is depicted in Figure 1, which shows the process of adding pseudo-labels and performing the training supervision.

### 2.2. Single-Person Walking Video Dataset

The Single-Person Walking Video Dataset (SPWVD) was collected from 860 preschool children recruited from Baoli Experimental Kindergarten and Binhu Kindergarten (Wuxi, Jiangsu Province, China). The study protocol was reviewed and approved by the Medical Ethics Committee of Jiangnan University (Approval No. JNU20230901IRB04). Written informed consent was obtained from the legal guardians of all participants, who voluntarily agreed to participate and to allow the use of the collected information under strict confidentiality.

Participant demographics were documented for cohort characterization and potential bias assessment (male/female: 426/434; age 5/6 years: 398/462) but were not used as model input features. A class-balanced annotated subset was constructed for leg alignment modeling and is summarized in Table 1 (87 samples per class). As this dataset was collected in a kindergarten-based screening context, the labels are intended for morphology-oriented gait/leg alignment recognition rather than clinical diagnosis; therefore, the cohort may naturally include both developmental (physiological) variations and potential pathological cases, and individuals with pronounced abnormalities should be referred for clinical assessment.

Video recording was conducted in an indoor corridor with sufficient lighting, minimal background noise, and no interference from other pedestrians. The camera was placed at the 20 m endpoint of the walking path, directly facing the subject, with the lens positioned 0.5 m above the ground. Each gait video lasted 15–20 s, and the camera’s field of view covered the entire scene, recording a complete 20 m out-and-back walk with clear and continuous footage.

In this study, we consider both binary and three-class classification tasks. For the binary task, the positive class is defined as abnormal gait, following the diagnostic description in Practical Diagnostics, which includes typical manifestations such as hesitation at gait initiation, foot dragging or excessive swing-foot height, asymmetric step length, step discontinuity, lateral deviation during walking, trunk swaying, and an overly wide stance.

For leg alignment-related gait categories, labels were defined based on objective anatomical alignment criteria described in Practical Diagnostics [24,25]. Specifically, genu varum (bow-legged alignment) is defined when the medial malleoli can naturally approximate in a neutral standing position while the intercondylar distance between the medial femoral condyles is >0. In contrast, genu valgum (knock-kneed alignment) is defined when the medial femoral condyles can naturally approximate while the intermalleolar distance between the medial malleoli is >0. Accordingly, the three classes are defined as Normal/Genu varum/Genu valgum; for the binary task, Abnormal includes genu varum and genu valgum, whereas Normal denotes physiologically normal alignment.

**Figure 2:**
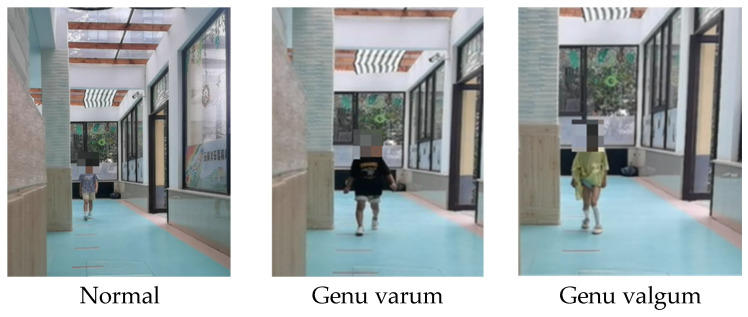
Normal, genu varum, genu valgum.

Gait classification and coding were performed collaboratively by senior professionals, including orthopedic surgeons and Ph.D.-level experts in sports science, all holding associate professor or higher academic titles. Two assessors independently provided initial labels following the unified criteria; discrepant or borderline cases were adjudicated by a third orthopedic surgeon with documented rationale to form the final ground-truth labels for training and evaluation. Since the original workflow did not preserve complete independent rater records in a format intended for statistical agreement analysis, Cohen’s kappa and related quantitative reliability metrics were not reported; this limitation is stated in the Discussion. Detailed class definitions and coding rules are provided in Table 1, and representative examples of the three-class setting are illustrated in Figure 2.

### 2.3. CASIA-B Video Dataset (CSBVD)

The CASIA-B dataset was used as unlabeled data to support semi-supervised learning [26]. Considering that CASIA-B consists of adult gait sequences captured under different conditions, we configured the unlabeled subset to reduce viewpoint-induced domain shift. Specifically, we sampled only the 0° and 180° viewpoints, which are consistent with the viewpoint geometry of SPWVD, where the camera was directly facing the subject (i.e., frontal/back views). This viewpoint alignment helps ensure that the unlabeled data primarily contributes generic gait motion regularization rather than introducing additional viewpoint confounding.

**Figure 3:**
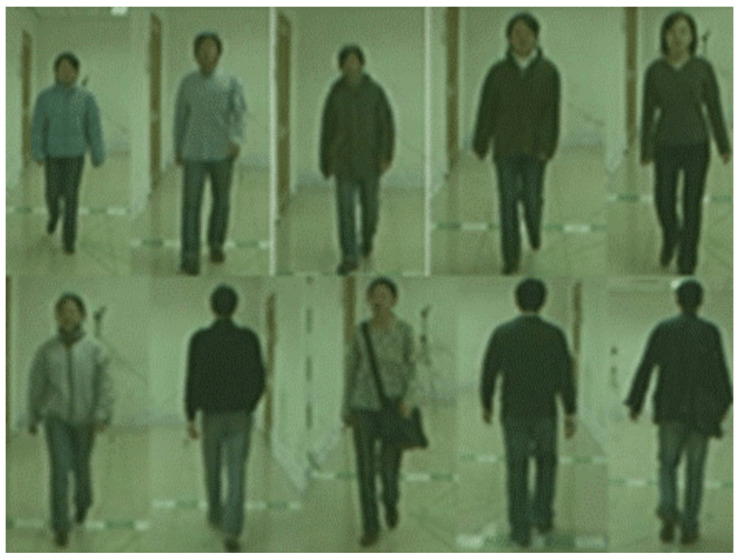
Gait images in CASIA-B at 0° and 180° viewing angles.

As shown in Figure 3, we randomly selected 364 video clips from 124 individuals walking at 0° and 180° viewpoints to form the unlabeled set. All clips were processed using the same preprocessing pipeline as SPWVD before being fed into the network.

To prevent data leakage and ensure unbiased evaluation, SPWVD was split at the subject level with a 64/16/20 train/validation/test ratio. Each child contributed exactly one full-length video and appeared in only one split, with no subject overlap across sets.

### 2.4. Mean Teacher Module (MTM)

MTM is a semi-supervised learning method designed to enhance the generalization capability of neural network models, particularly in scenarios where labeled data are scarce. Using both labeled and unlabeled data, the method addresses the challenge of limited annotated datasets, which are common in many fields, such as medical imaging and video analysis.

#### 2.4.1. Teacher and Student Network Model

The MTM framework comprises two neural network models: a teacher model and a student model. The parameters of the teacher model are not directly learned through backpropagation. Instead, they are updated using the Exponential Moving Average (EMA) of the student model’s parameters. This process allows the teacher model to serve as a stable reference for the student during training, smoothing the learning process, and reducing overfitting to the training data. The student model, on the other hand, is trained in a conventional manner. It learns directly from the labeled data and adjusts its parameters through a standard gradient descent. During each iteration, the predictions of the student model are compared with the pseudo-labels of the teacher model for the unlabeled data. This consistency regularization ensures that the student network’s outputs are consistent with the teacher’s outputs, even for unlabeled examples, thus improving its generalization capabilities.

By promoting consistency between the predictions of the student model and the pseudo-labels of the teacher model on the unlabeled data, the MTM framework leads to more generalized feature representations. This consistency loss acts as a regularizer, improving the model’s ability to learn from incomplete or imprecise labels, thus mitigating overfitting to the labeled set. The combination of EMA updates for the teacher model and the 3D ResNet backbone architecture results in a more stable training process, which is crucial for handling the high-dimensional nature of 3D data.

#### 2.4.2. Features Aggregation and Hierarchical Operations

Feature aggregation is achieved by integrating the pooled blocks into higher-level representations, which capture the essential features of each block and help preserve critical spatiotemporal information. This module employs hierarchical operations across multiple levels, with each level utilizing different clustering sizes or strides. The aggregated features are subsequently passed through connected layers for further classification and computation [27].

#### 2.4.3. Semi-Supervised Learning

In the first training phase (pre-training), labeled data is used to perform supervised learning, resulting in an initial model, which then employs semi-supervised learning to predict pseudo-labels for the unlabeled data. In the second training phase, labeled and unlabeled data with pseudo-labels are mixed to form an extended training dataset. This updated dataset is used to train the model, during which the parameters and weights of the teacher model are updated using the EMA to better adapt to both labeled and pseudo-labeled data [18,27]. The objective function for this modeling process is shown in Equations (1)–(3).

(1) $$\begin{array}{c} minL\left(\theta ,\theta^{\prime }\right)\\ s.t.L\left(\theta ,\theta^{\prime }\right)=\overset{n}{\underset{i=0}{\sum }}\lambda L_{S_{i}}\left(\theta \right)+\overset{k}{\underset{i=0}{\sum }}\left(1-\lambda \right)J_{i}\left(\theta ,\theta^{\prime }\right) \end{array}$$

(2) $$L_{S_{i}}\left(\theta \right)=\mathrm{CrossEntropy}\left(S\left(X_{i},\theta \right),Y_{i}\right)$$

(3) $$J_{i}\left(\theta ,\theta^{\prime }\right)=E{||S\left(Z_{i},\theta \right)-T\left(Z_{i},\theta^{\prime }\right)||}_{2}^{2}$$

Here, n and k denote the numbers of labeled and unlabeled samples, respectively. *L_S_*(*θ*) is the supervised loss on labeled data, implemented as the cross-entropy between the student prediction and the ground-truth label. For a labeled sample *X_i_*, we denote the student output as *S_i_* = *S*(*X_i_, θ*), which is converted to a class-probability vector *p_s_* ∈ *R^C^* via the softmax function, where C is the number of classes [28]. Accordingly, the cross-entropy loss is computed on these normalized probabilities (Equations (4) and (5)). Specifically, we adopt soft pseudo-labels to calculate the consistency loss. By minimizing the squared 2-norm distance between the probability distributions of the teacher and student, the model preserves prediction uncertainty, which is crucial for mitigating confirmation bias. This strategy prevents the student from overfitting to potential erroneous ‘one-hot’ targets during the early phases of training.

(4) $$p_{i}=\frac{exp\left\{l_{i}\right\}}{\sum_{j=1}^{C}\exp\left\{l_{j}\right\}}$$

where *l_i_* is the logit of the *i*th class. The CE loss can consequently be calculated as

(5) $$\mathrm{CrossEntropy}\left(S,Y_{i}\right)=\sum_{i=1}^{C}-Y_{i}\log\;p_{i}\left(S_{i}\right)$$

where *T* (*Z_i_*, *θ*^′^) stands for the teacher model, *S_i_*(*Z_i_*, *θ*) stands for the student model with respect to input *Z_i_*, and *θ*^′^ and *θ* represent the weights of the teacher and student models, respectively. *J_i_*(*θ*, *θ*^′^) calculates the expectation value of the square of the 2-norm in the student and teacher model based on their prediction over the same unlabeled dataset. And *λ* is an increasing weighting coefficient that controls the trade-off between supervised and unsupervised losses.

#### 2.4.4. Coefficient Update

In the Mean Teacher model, the parameters of the student network are updated via gradient descent, as shown in Equation (6). Meanwhile, the teacher network tracks the student network using an Exponential Moving Average. Specifically, after each training iteration, the teacher’s parameters *θ′* are updated by computing a weighted average between its previous parameters and the current parameters of the student network, as defined in Equations (6) and (7).

(6) $$\theta =\theta -\gamma \cdot \frac{\partial L}{\partial \theta }$$

(7) $$\theta^{\prime }=\alpha \theta^{\prime }+\left(1-\alpha \right)\theta \;\;\;$$

where *α* (typically close to 1, e.g., 0.99) is the update rate. This mechanism allows the teacher model to gradually incorporate the student’s learned parameters over time, effectively smoothing out fluctuations and providing more stable targets for the student. The EMA ensures that the teacher model captures the historical progression of the student model’s parameters, thereby enhancing generalization by maintaining consistent and reliable guidance during training. Algorithm 1 systematically delineates each step of the proposed methodology in detail. We set the confidence bound to be 0.8, that is, any pseudo-label giving higher confidence will be considered as efficient.
**Algorithm 1:** MTM Implemented Algorithm
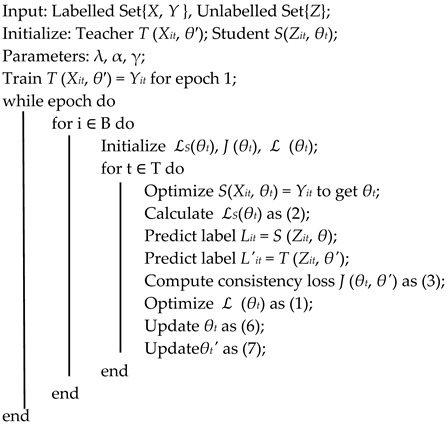


### 2.5. 3D ResNet-18 and Spatial Hierarchical Pooling Module (SHPM)

**Figure 4:**
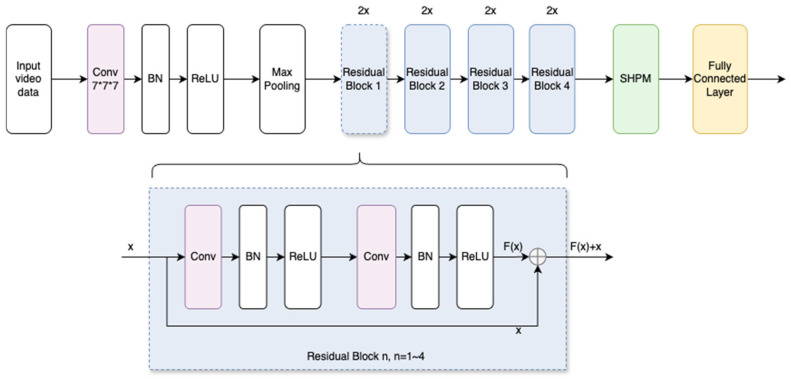
3DResNet-18 with SHPM.

A 3D ResNet-18 architecture is employed as the backbone for both teacher and student networks, as it is particularly well suited for tasks involving 3D data, such as volumetric medical imaging or video-based activity recognition [29]. The 3D ResNet efficiently captures spatiotemporal features by applying 3D convolutions, allowing the model to learn both spatial and temporal dependencies in the data. Figure 4 shows detailed information on its composition. In the context of MTM, this architecture benefits from the semi-supervised framework by extracting more robust features from both labeled and unlabeled 3D data.

We update the final step by changing the extraction method to our introduced SHPM to reach the balance between the preservation of information and the efficiency of the calculation. According to the proposed HPM for better matching the person Re-Identification (ReID) from Fu [30], due to the similarity between the size of the walking figure and the examples used in it, we update the dimensions to a more complicated step to ensure that the model will not lose any useful information. Especially efficient for a limited amount of video data input, SHPM can separate each frame and extract the corresponding features individually.

SHPM processes an input tensor with dimensions *B* × *C* × *T* × *H* × *W*, where *B* represents the batch size, *C* denotes the number of channels, *T* corresponds to the temporal dimension and *H* and *W* represent the height and width, respectively. The input spatial data will be divided into blocks that correspond to different spatial bins or time intervals, denoted as *H_bins_* ∗ *W_bins_* (Equation (8)).

(8) $$H_{\mathrm{bins}\;}=\frac{H}{H_{\mathrm{Size}\;}},W_{\mathrm{bins}\;}=\frac{W}{W_{\mathrm{Size}\;}}$$

A fundamental operation in deep learning is used to reduce spatial dimensions while retaining the features of the original input data (Equation (9)). SHPM actually combines the two most commonly used types of pooling: max pooling (Equation (10)) to emphasize the most prominent features, and average pooling (Equation (11)) to keep the average strength across the region.

(9) $$\mathrm{substitute}\;X=X_{b,c,t,i\times H_{\mathrm{size}\;}+h,j\times W_{\mathrm{size}\;}+w}$$

(10) $$\mu_{b,c,t,i,j}=\underset{h}{max}\underset{w}{max}X$$

(11) $$\eta_{b,c,t,i,j}=\left(\frac{1}{H_{\mathrm{size}}\times W_{\mathrm{size}}}\right)\overset{H_{\mathrm{size}}-1}{\underset{h=0}{\sum }}\overset{W_{\mathrm{size}}-1}{\underset{w=0}{\sum }}X$$

**Figure 5:**
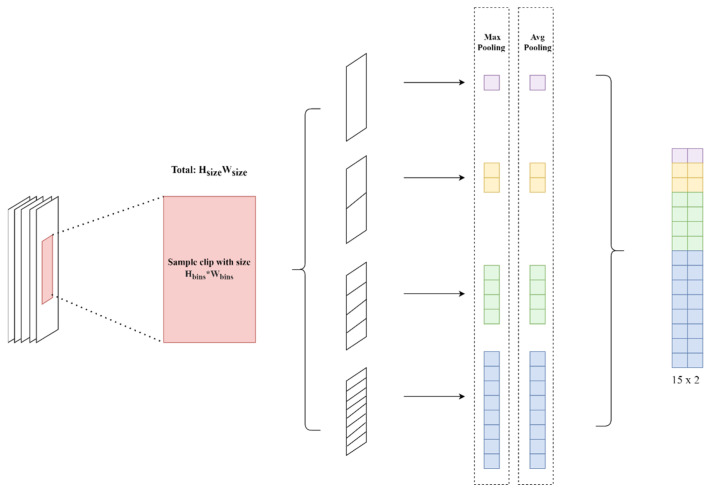
SHPM.

*η_b,c,t,i,j_* is the average value, with the indices *i* and *j*; *µ_b,c,t,i,j_* is the maximum value for the indicated region. The entire SHPM process is shown in Figure 5.

## 3. Results

### 3.1. Model Training

Based on the task and available hardware, the input is batched into mini-batches of 16 samples to utilize computational resources and improve training efficiency. From each video frame (resolution 2560 × 1440), a 224 × 224 region is positioned in the center to reduce computation and suppress irrelevant noise while retaining salient content. The videos are then decomposed into image frames at a fixed frame rate and grouped into clips of 16 consecutive frames so that the model can capture temporal continuity and dynamics [31]. We use the Adam optimizer (learning rate *η* = 1 × 10^−4^, *β*_1_ = 0.9, *β*_2_ = 0.999), with a batch size of 16 and a total of 100 epochs; the supervised loss is cross-entropy. To curb overfitting, we adopt early stopping (training terminates if the validation metric shows no improvement for 10 consecutive epochs) and a Reduce-on-Plateau scheduler (the learning rate is halved if the validation loss does not decrease for 5 consecutive epochs) [32].

To accommodate the semi-supervised setting, we employ a Mean Teacher framework: the teacher parameters are updated via EMA (*α* = 0.99), initialized with *θ′* ← *θ*. For unlabeled samples, we use both a consistency loss and a pseudo-label loss with a confidence threshold *τ* = 0.8; the consistency weight *λ* increases from 0 to 1.0 over the first 30 epochs. For data augmentation, the teacher branch uses weak augmentation (center crop/light random crop, horizontal flip), while the student branch uses strong augmentation (random crop, color jitter, Gaussian noise, random erasing, temporal jitter, and speed perturbation). At test time, we apply center crop only and do not use test time augmentation. Unless otherwise noted, the results are reported as the mean ± standard deviation of five random seeds.

The confidence threshold τ serves as a quality gate for selecting reliable pseudo-labels from the unlabeled set. To stabilize semi-supervised optimization, we apply a ramp-up schedule to the consistency weight λ, increasing it linearly from 0 to 1.0 during the first 30 epochs. This allows the model to first establish a discriminative representation from the labeled pediatric data and then progressively leverage the unlabeled CASIA-B clips.

The dataset is divided into three subsets at the subject level with no subject overlap across splits: 64% for training, 16% for validation, and 20% for testing (following common practice) [33]. The training/validation sets contain both labeled and unlabeled data, while the test set contains only labeled data. In addition, 364 and 324 unlabeled CASIA-B clips were sampled for the training and validation sets, respectively; the unlabeled clips were taken from viewpoints 0° and 180°, matching the viewpoint geometry of our in-house acquisition (frontal/back views). The labeled pediatric SPWVD contains 860 clips (one full-length video per subject), and the breakdown of class labels is provided in Table 2. For fair comparison, all baseline models were trained with the same data preprocessing and optimization protocol as AGRM (clip length, input crop, batch size, optimizer, learning-rate schedule, epochs, and early stopping).

### 3.2. Simulation System

To test the effectiveness of the proposed model and adapt MTM training, the program was solved with Python. Detailed information is provided in Table 3.

### 3.3. Experiments Analysis

Due to the imbalanced class distribution in the binary screening setting (Normal vs. Abnormal), we report precision and recall as complementary evaluation metrics. Precision measures the proportion of the predicted abnormal samples that are truly abnormal, while recall reflects the proportion of the true abnormal samples that are correctly identified [34]. These metrics are defined as follows:

(12) $$\mathrm{Precision}\;=\frac{TP}{TP+FP}$$

(13) $$\mathrm{Recall}\;=\frac{TP}{TP+FN}$$

Binary task definition and thresholding: For the binary task, we define Abnormal as the positive class, where Abnormal merges genu varum and genu valgum, and Normal denotes physiologically normal alignment. At the test time, binary decisions are obtained from the predicted probability of Abnormal using a fixed threshold of 0.5. Note that the pseudo-label confidence threshold *τ* = 0.8 is used only during training to select unlabeled samples in the Mean Teacher framework and is not used for test time decision-making.

To provide a more clinically interpretable breakdown of error types under class imbalance, we additionally report the confusion matrix (TP/FP/FN/TN) and derived metrics including sensitivity (Recall), specificity (*TN*/(*TN* + *FP*)), PPV (*TP*/(*TP* + *FP*)), and NPV (*TN*/(*TN* + *FN*)) for the binary task, where TP, FP, FN, and TN denote true positives, false positives, false negatives, and true negatives, respectively. Moreover, we report the precision–recall (PR) curve and PR-AUC as threshold-independent measures that are informative when the prevalence of abnormal gait in the dataset may differ from that in real-world screening scenarios. In the current revision, we specify these metrics for completeness; however, their numerical reporting is not included because the original workflow did not retain the full test time probability outputs required for post hoc computation (e.g., PR curves). This limitation is acknowledged in Section 4.5, and we will address it by re-running inference with fixed seeds and reporting the full evaluation summary in a subsequent revision.

In the multiclass classification tasks, the model performance is evaluated using the accuracy, macro-precision, macro-recall, and macro-F1 score. These metrics provide insights from different perspectives on the effectiveness of the model [35], as defined in Equations (14)–(16).

(14) $${\mathrm{Macro}\;}_{P}=\frac{1}{n}\sum_{i=1}^{n}P_{i}$$

(15) $${\mathrm{Macro}\;}_{R}=\frac{1}{n}\sum_{i=1}^{n}R_{i}$$

(16) $${\mathrm{Macro}\;}_{F_{1}}=\frac{2*{\mathrm{Macro}\;}_{P}*{\mathrm{Macro}\;}_{R}}{{\;\mathrm{Macro}\;}_{P}+{\;\mathrm{Macro}\;}_{R}}$$

#### 3.3.1. Model Performance Evaluation

The performance of the proposed AGRM was comprehensively evaluated in binary and multiclass classification settings, and the results are summarized in Table 4.

In binary (2C) and three-class (3C) settings, we report the precision and macro-averaged (MA) precision, recall, and F1 score. In general, the differences between the training, validation, and test sets are small, indicating that there are no obvious overfits and good generalizability. Meanwhile, the 2C setting outperforms the 3C setting, as expected given the increased classification difficulty with more classes. The MA metrics closely track accuracy, suggesting a balanced recognition across classes.

#### 3.3.2. AGRM and Baselines: Test Set Experimental Results

To assess performance on the held-out test set, we evaluated AGRM against 3D ResNet, R(2 + 1)D, C3D, and UniFormer in binary and three-class settings. All metrics are computed on the test partition only, with no validation or training data used for evaluation. AGRM achieves the highest accuracy and macroaveraged precision/recall/F1 across all splits, indicating stable recognition under the variations present in the test data (e.g., viewpoint, clothing, background). The detailed results are summarized in Table 5.

#### 3.3.3. Ablation Experiment Results

Ablation studies are conducted to assess the contribution of key components and to validate the modeling design. Specifically, we remove the Mean Teacher Module and the Spatial Hierarchical Pooling Module in a controlled manner. The ablation results are reported in Table 6. These ablations serve as controlled baselines corresponding to (i) Mean Teacher without SHPM and (ii) SHPM without Mean Teacher, evaluated under the same training protocol.

### 3.4. Model Visualization

**Figure 6:**
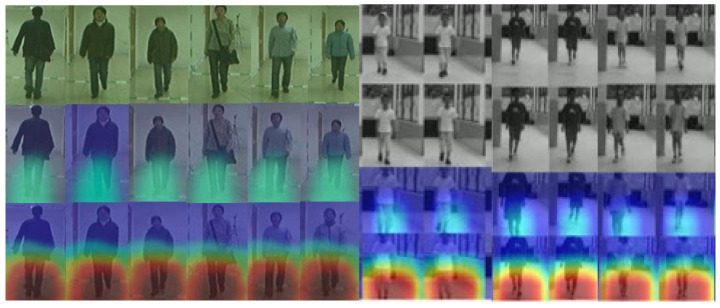
Grad-CAM visualization.

Using Grad-CAM, we visualize class-discriminative regions of AGRM (Figure 6). Across the examples shown, the model’s attention is mainly concentrated on the lower extremities, particularly around the knee region, when distinguishing gait patterns related to leg alignment. This qualitative observation is consistent with clinical reasoning for genu valgum and genu varum, where frontal-plane knee alignment and associated lower limb kinematics are key cues.

For reproducibility, Grad-CAM maps were generated using a unified protocol: we compute gradients with respect to the logit of the predicted class and use the last convolutional block of the 3D backbone as the target layer; the resulting activation maps are min–max normalized within each clip and overlaid on the corresponding RGB frames using the same colormap and transparency settings across all classes. We emphasize that Grad-CAM provides a qualitative explanation of model behavior rather than a causal or diagnostic localization guarantee. In some borderline or noisy cases (e.g., mild deformity, partial occlusion, or motion blur), attention may partially drift to non-informative regions such as clothing edges or background structures, which may contribute to misclassification. These failure modes motivate future work on more robust region-of-interest constraints (e.g., key point-guided attention) and multimodal sensing to improve interpretability under challenging conditions.

## 4. Discussion

### 4.1. Comparison Between Binary and Multiclass Classification

The higher accuracy observed in the binary task is largely attributable to the improved separability when abnormal gait is collapsed into a simple Normal vs. Abnormal dichotomy. By contrast, in the more fine-grained three-class setting (Normal, Genu valgum, and Genu varum), interclass separation can be limited, while intraclass variability remains substantial [36]. Biomechanical evidence suggests that gait alterations associated with valgus/varus alignment often manifest as modest shifts in frontal-plane knee kinematics and knee adduction mechanics, which may be particularly subtle in mild cases [37]. The differences tend to become more evident only with greater deformity or in the presence of symptomatic knee osteoarthritis; accordingly, when classes are partitioned by finer criteria such as severity, the Bayes error is expected to increase [38]. This overlap across closely related categories has been noted in cohorts of genu valgum/varum as well as in the knee osteoarthritis gait literature, where kinematic and kinetic deviations—such as reduced tibial rotation excursion, alterations in the screw-home mechanism, and varus–valgus thrust—typically scale with the degree of malalignment and overall disease burden [39].

A second driver is data and annotation economy. Multiclass recognition requires sufficient well-labeled samples per class [40]. In clinical gait datasets, expert annotation is scarce and subject to inter-rater variability; mild cases are particularly ambiguous. Empirically, label noise degrades performance disproportionately in multiclass settings because errors disperse the probability mass between multiple neighboring classes, flattening the decision margins [41]. Recent surveys and experiments in medical imaging and multiclass vision confirm that deep networks easily fit noisy labels and that accuracy drops as the noise increases, especially with more classes [42].

Third, nuisance variability (walking speed, fatigue state, treadmill vs. overground, footwear, camera/viewpoint) widens within-class dispersion for spatiotemporal and joint angle characteristics [43]. The gait variability literature shows stride-to-stride fluctuations and speed-dependent shifts in kinematics and kinetics across populations; such covariate shifts blur multiclass decision boundaries unless explicitly modeled. In contrast, binary grouping absorbs a lot of this variability [44]. Finally, from a learning theory perspective, multi-class problems typically require more data and a stronger inductive bias than binary ones [45]; increasing the number of classes with fixed data inflates the variance of estimation and reduces margins unless compensated for by architecture or loss design. Formal and empirical analyses of multiclass hardness echo this observation [46].

Implications and remedies: Given that valgus/varus severity is ordinal, reframing multiclass recognition as ordinal regression leverages intrinsic order to enlarge the effective margins between neighboring severities (e.g., CORAL/CORN), which has improved rank-consistent predictions in deep models [47]. Complementarily, metric learning losses with explicit angular margins (e.g., ArcFace) can tighten intraclass clusters and separate fine-grained classes [48]. When class frequencies are uneven, cost-sensitive criteria (e.g., Focal Loss; Class-Balanced Loss based on effective number of samples) help counter long-tailed distributions and hard-negative dominance. These choices are particularly suitable when combined with the semi-supervised training already used in this work [49].

### 4.2. Reasons for Outperforming Baseline Models

AGRM outperforms baseline approaches in cross-dataset testing due to the synergy between its architectural design and semi-supervised training strategy. This combination mitigates the domain shift while preserving fine-grained pathological gait cues. The Mean Teacher framework improves generalization under heterogeneous capture conditions, whereas SHPM retains multiscale clinically significant motion details. Together, they establish robust and transferable decision boundaries without additional labeling cost.

#### 4.2.1. Mean Teacher Framework: Robust Generalization from Unlabeled Data

The Mean Teacher framework leverages unlabeled gait sequences through consistency regularization, maintaining prediction stability under perturbations, and updates the teacher network via an Exponential Moving Average [50]. This process reduces representation variance and mitigates confirmation bias in pseudo-labels, anchoring decision boundaries to domain-invariant structures rather than dataset-specific noise (e.g., viewpoint, apparel, walking speed). Consequently, it substantially improves generalization and cross-dataset transfer, particularly in label-scarce scenarios [51].

#### 4.2.2. SHPM: Multiscale Spatial Sensitivity with Efficient Aggregation

The SHPM preserves subtle kinematic signals while maintaining computational efficiency. By aggregating features across multiple spatial receptive fields, it captures both global configurations (e.g., pelvic tilt, stride width) and local discriminators (e.g., valgum/varum asymmetry, foot progression angle), forming scale-invariant representations. These representations remain robust to variations in pose, scale, and resolution, effectively overcoming the limitations of single-scale pooling to retain fine-grained details [52,53].

#### 4.2.3. Test Set Performance Comparison: AGRM vs. Baseline Models

MTM and SHPM are highly complementary: MTM, through semi-supervised learning driven by consistency, extracts domain-invariant spatiotemporal characteristics [54], while SHPM enhances feature representations with global stability and local pathological sensitivity. This globally robust + locally precise design enables AGRM to maintain semantic stability while detecting fine-grained pathological differences under diverse acquisition conditions [55]. In contrast, conventional video modeling networks such as 3D ResNet, R(2 + 1)D, and C3D rely heavily on large-scale labeled supervision and tend to fit too much into the training domain [56]. When exposed to out-of-domain data with different scenes, lighting, or viewpoints, their decision boundaries drift, reducing interclass separability and causing simultaneous drops in accuracy and recall [57,58].

The results of the cross-dataset evaluation (Table 5) show that AGRM consistently maintains a performance advantage in all test domains, with significantly higher mean accuracy and macro-averaged F1 scores than the baselines. This demonstrates that AGRM is more effective in addressing the combined challenges of limited labeled data, domain change, and fine-grained pathological recognition, an advantage stemming not only from its architectural innovations, but also from the synergy between unlabeled data utilization and multiscale feature fusion.

### 4.3. Analysis of Module Contributions

Ablation studies (as shown in Table 6) indicate that MTM and SHPM provide complementary gains in precision and robustness between datasets. Removing MTM yields the largest drop in label-scarce regimes under dataset transfer, consistent with its role in reducing representation variance and curbing pseudo-label confirmation bias. Removing SHPM particularly alters the sensitivity to fine-grained anomalies and degrades transfer across view or resolution changes, aligning with its design goal of preserving multiscale spatial evidence. The two modules reinforce each other: SHPM supplies stable multiscale cues, while MTM aligns predictions across stochastic augmentations of those cues, suppressing cross-scale noise amplification. This synergy leads to (1) a better calibration and lower variance between random seeds, (2) a stronger resilience to occlusion and temporal jitter, and (3) a greater recall of clinically relevant abnormal patterns that single-scale purely supervised systems often miss.

### 4.4. Clinical Relevance

GradCAM visualizations reveal that AGRM consistently cares for clinically relevant anatomical regions such as the knee joint, lower leg, and surrounding soft tissues, aligning closely with clinician focus areas in the assessment of gait pathology [58]. Similarly to recent work evaluating explainable AI approaches for detecting gait abnormalities in patients with cerebral palsy, our Grad-CAM analysis confirms that AGRM is based on clinically relevant biomechanical features, such as deviations of the valgus/varus, stride asymmetry, and angle of progression of the foot, rather than extraneous signals.

This explainability enables practical implementation in early pediatric screening and postural evaluation within community or school settings. The low-cost video-enabled design supports the timely identification of mild abnormalities for corrective interventions and facilitates the referral of severe cases. In addition, AGRM’s interpretable outputs provide an accessible complement to traditional gait laboratories and encourage coordinated care across orthopedics, pediatrics, sports medicine, and rehabilitation disciplines.

### 4.5. Limitations and Future Work

Despite the strong overall performance, the multiclass accuracy of AGRM is constrained by several factors. First, the diversity of abnormal gait samples is still limited, which may reduce intra-class and inter-class variability and hinder the learning of more generalizable features. Second, semi-supervised training inevitably introduces potential label noise from pseudo-labeled unlabeled data. Third, leg alignment patterns (normal/varum/valgum) can lie on a continuous spectrum, and borderline cases may exhibit subtle visual differences that are difficult to separate reliably from RGB videos alone.

In addition, while we define confusion matrix-based clinical metrics and PR-AUC for completeness, we did not include their numerical reporting in this revision because the original experimental workflow did not preserve the complete test time probability outputs required for post hoc computation. We will address this by re-running inference with fixed seeds and providing the full evaluation summary in a subsequent revision.

Future work will explore incorporating multimodal inputs (e.g., depth or inertial signals), integrating uncertainty estimation to improve pseudo-label quality, and investigating hybrid CNN–Transformer backbones for richer spatiotemporal representations. Moreover, because pseudo-labeling in semi-supervised learning may affect probability calibration, we focus on discrimination metrics in this work and do not conduct a dedicated calibration study; calibration-oriented evaluation and post hoc calibration (e.g., temperature scaling) will be explored in future work.

## 5. Conclusions

The early identification of pediatric leg alignment-related gait abnormalities can support timely triage and referral, helping reduce the risk of prolonged biomechanical maladaptation. In this work, we proposed AGRM, a video-based abnormal gait recognition framework that integrates a 3D ResNet backbone with a Mean Teacher (MTM) semi-supervised training strategy and a Spatial Hierarchical Pooling Module (SHPM) to capture multi-level spatiotemporal gait cues.

Experiments on our hybrid dataset show that AGRM consistently outperforms representative supervised baselines in both the three-class task (Normal/Genu varum/Genu valgum) and the binary screening task (Normal vs. Abnormal). Specifically, AGRM yields a higher accuracy and macro-averaged F1 (MA F1) in both settings, and the ablation results confirm that both MTM and SHPM contribute meaningfully to the observed gains.

Importantly, we position AGRM as a screening support tool rather than a diagnostic system. Its intended use is to assist triage in community or school scenarios by flagging gait patterns that may warrant further orthopedic/pediatric assessment, enabling low-cost and scalable early screening support.

This study has limitations. First, our labels are morphology-oriented and do not constitute clinical diagnosis, severity grading, or treatment decisions. Second, domain shift between pediatric videos and public datasets may affect generalization under unconstrained real-world conditions. Third, some screening-oriented metrics that require probability outputs (e.g., PR-AUC) depend on logging/inference settings and will be fully standardized in future evaluations. Future work will expand the pediatric cohort and acquisition diversity, strengthen domain adaptation and calibration for screening, and further enhance interpretability with quantitative sanity checks (e.g., region occlusion) to support clinically plausible model behavior.

## Figures and Tables

**Table 1:** Classification and encoding.

	2-Class		3-Class		
	Normal	Abnormal	Normal	Genu Varum	Genu Valgum
Encoding	1	2	11	21	22

**Table 2:** Data composition of the merge dataset.

	Normal	Genu Valgum	Genu Varum	Unlabeled
Training set	87	87	87	291
Validation set	21	21	21	73
Testing set	58	57	57	–

**Table 3:** Experimental environment setup.

Settings	Details
CPU	Model: i7-7700, Frequency: 3.6 GHz, 4 cores 8 threads 64 bits
GPU	NVIDIA GeForce GTX1060, Memory: 6 GB
Memory	8 GB
Operation System	Windows 10 professional
Coding Language	Python 3.7

**Table 4:** Overall performance of AGRM on train/validation/test (2-class and 3-class).

	Accuracy	MA Precision		MA Recall		MA F1 Score
	3C	2C	3C	2C	3C	3C
Training	0.725	0.834	0.734	0.853	0.732	0.732
Validation	0.715	0.832	0.715	0.815	0.725	0.725
Testing	0.705	0.803	0.721	0.792	0.715	0.718

**Table 5:** Test set comparison: AGRM vs. baselines (2-class and 3-class).

	Accuracy	MA Precision		MA Recall		MA F1 Score
	3C	2C	3C	2C	3C	3C
R(2 + 1)D	0.680	0.774	0.660	0.768	0.650	0.660
UniFormer	0.691	0.752	0.690	0.815	0.725	0.725
C3D	0.624	0.737	0.612	0.685	0.643	0.639
3DResNet	0.616	0.682	0.674	0.668	0.656	0.671
AGRM	0.705	0.803	0.721	0.792	0.715	0.718

**Table 6:** Ablation on MTM and SHPM (test set).

	Accuracy	MA Precision		MA Recall		MA F1 Score
	3C	2C	3C	2C	3C	3C
Remove SHPM	0.614	0.665	0.585	0.623	0.556	0.571
Remove MTM	0.663	0.745	0.681	0.714	0.644	0.668
AGRM	0.705	0.803	0.721	0.792	0.715	0.718

## Data Availability

The original contributions presented in this study are included in the article. Further inquiries can be directed to the corresponding author.

## References

[1] Li Y.B., Jiang T.X., Qiao Z.H., Qian H.J. (2007). General methods and development actuality of gait recognition. 2007 International Conference on Wavelet Analysis and Pattern Recognition.

[2] Lehnert–Schroth C., Auner–Gröbl P. (2021). Dreidimensionale Skoliosebehandlung: Atmungs–Orthopädie System Schroth.

[3] Brouwer G.M., van Tol A., Bergink A.P., Belo J.N., Bernsen R.M.D., Reijman M., Pols H.A.P., Bierma-Zeinstra S.M.A. (2007). Association between valgus and varus alignment and the development and progression of radio- graphic osteoarthritis of the knee. Arthritis Rheum..

[4] Moisio K., Chang A., Eckstein F., Chmiel J.S., Wirth W., Almagor O., Prasad P., Cahue S., Kothari A., Sharma L. (2011). Varus–valgus alignment: Reduced risk of subsequent cartilage loss in the less loaded compartment. Arthritis Rheum..

[5] Sharma L., Song J., Felson D.T., Cahue S., Shamiyeh E., Dunlop D.D. (2001). The role of knee alignment in disease progression and functional decline in knee osteoarthritis. JAMA.

[6] Soheilipour F., Pazouki A., Mazaherinezhad A., Yagoubzadeh K., Dadgostar H., Rouhani F. (2020). The prevalence of genu varum and genu valgum in overweight and obese patients: Assessing the relationship between body mass index and knee angular deformities. Acta Biomed. Atenei Parm..

[7] Shetty G.M., Mullaji A., Bhayde S., Nha K.W., Oh H.K. (2014). Factors contributing to inherent varus alignment of lower limb in normal Asian adults: Role of tibial plateau inclination. Knee.

[8] Müller J., Müller S., Baur H., Mayer F. (2013). Intra-individual gait speed variability in healthy children aged 1–15 years. Gait Posture.

[9] Purish S.V., Lobachev M.V. (2023). Gait recognition methods in the task of biometric human identification. Her. Adv. Inf. Technol..

[10] Wu Z., Zhong M., Jiang X., Shen B., Zhu J., Pan Y., Dong J.D., Yan J., Xu P.Y., Zhang W.B. (2020). Can quantitative gait analysis be used to guide treatment of patients with different subtypes of Parkinson’s disease?. Neuropsychiatr. Dis. Treat..

[11] Silva J., Atalaia T., Abrantes J., Aleixo P. (2024). Gait biomechanical parameters related to falls in the elderly: A systematic review. Biomechanics.

[12] Wan C., Wang L., Phoha V.V. (2018). A survey on gait recognition. ACM Comput. Surv..

[13] Sepas-Moghaddam A., Etemad A. (2022). Deep gait recognition: A survey. IEEE Trans. Pattern Anal. Mach. Intell..

[14] Wang X., Yan W.Q. (2020). Human gait recognition based on frame-by-frame gait energy images and convolutional long short–term memory. Int. J. Neural Syst..

[15] Li K., Wang Y., Gao P., Song G., Liu Y., Li H., Qiao Y. (2022). UniFormer: Unified transformer for efficient spatiotemporal representation learning. arXiv.

[16] Tran D., Bourdev L., Fergus R., Torresani L., Paluri M. (2015). Learning spatiotemporal features with 3D convolutional networks. Proceedings of the IEEE International Conference on Computer Vision.

[17] Li K., Wang Y., Zhang J., Gao P., Song G., Liu Y. (2023). UniFormer: Unifying convolution and self-attention for visual recognition. IEEE Trans. Pattern Anal. Mach. Intell..

[18] Yang Q., Wei X., Wang B., Hua X.S., Zhang L. (2021). Interactive self-training with mean teachers for semi-supervised object detection. Proceedings of the IEEE/CVF Conference on Computer Vision and Pattern Recognition.

[19] Sun Y., Long H., Feng X., Nixon M. (2024). GaitASMS: Gait recognition by adaptive structured spatial representation and multi–scale temporal aggregation. Neural Comput. Appl..

[20] Wang L., Liu B., Wang B., Yu F. (2023). GAITMM: Multi-granularity motion sequence learning for gait recognition. 2023 IEEE International Conference on Image Processing (ICIP).

[21] Lian X., Pang Y., Han J., Pan J. (2021). Cascaded hierarchical atrous spatial pyramid pooling module for semantic segmentation. Pattern Recognit..

[22] Pinčić D., Sušanj D., Lenac K. (2022). Gait recognition with self-supervised learning of gait features based on vision transformers. Sensors.

[23] Wren T.A., Gorton G.E., Ounpuu S., Tucker C. (2011). Efficacy of clinical gait analysis: A systematic review. Gait Posture.

[24] Pirker W., Katzenschlager R. (2017). Gait disorders in adults and the elderly: A clinical guide. Wien. Klin. Wochenschr..

[25] Thomason P., Tan A., Donnan A., Rodda J., Graham H.K., Narayanan U. (2018). The Gait Outcomes Assessment List (GOAL): Validation of a new assessment of gait function for children with cerebral palsy. Dev. Med. Child Neurol..

[26] Tan D., Huang K., Yu S., Tan T. (2006). Efficient night gait recognition based on template matching. 18th International Conference on Pattern Recognition (ICPR).

[27] Singh A., Rana A.J., Kumar A., Vyas S., Rawat Y. (2024). Semi–supervised active learning for video action detection. Proceedings of the AAAI Conference on Artificial Intelligence.

[28] Zhao B., Cui Q., Song R., Qiu Y., Liang J. (2022). Decoupled knowledge distillation. Proceedings of the IEEE/CVF Conference on Computer Vision and Pattern Recognition.

[29] She Z., Marzullo A., Destito M., Spadea M., Leone R., Anzalone N., Steffanoni S., Erbella F., Ferreri A., Ferrigno G. (2023). Deep learning–based overall survival prediction model in patients with rare cancer: A case study for primary central nervous system lymphoma. Int. J. Comput. Assist. Radiol. Surg..

[30] Fu Y., Wei Y., Zhou Y., Shi H., Huang G., Wang X., Yao Z., Huang T. (2019). Horizontal pyramid matching for person re-identification. Proceedings of the AAAI Conference on Artificial Intelligence.

[31] Dave I.R., Rizve M.N., Chen C., Shah M. (2023). TimeBalance: Temporally-invariant and temporally-distinctive video representations for semi-supervised action recognition. Proceedings of the IEEE/CVF Conference on Computer Vision and Pattern Recognition.

[32] Croitoru F.A., Ristea N.C., Ionescu R.T., Sebe N. (2025). Learning rate curriculum. Int. J. Comput. Vis..

[33] Bengio Y. (2012). Practical recommendations for gradient–based training of deep architectures. Neural Networks: Tricks of the Trade.

[34] Kynkäänniemi T., Karras T., Laine S., Lehtinen J., Aila T. (2019). Improved precision and recall metric for assessing generative models. Advances in Neural Information Processing Systems.

[35] Tharwat A. (2021). Classification assessment methods. Appl. Comput. Inform..

[36] Barrios J.A., Heitkamp C.A., Smith B.P., Sturgeon M.M., Suckow D.W., Sutton C.R. (2016). Three-dimensional hip and knee kinematics during walking, running, and single-limb drop landing in females with and without genu valgum. Clin. Biomech..

[37] Ganesan B., Fong K.N.K., Luximon A., Al-Jumaily A. (2016). Kinetic and kinematic analysis of gait pattern of 13-year-old children with unilateral genu valgum. Eur. Rev. Med. Pharmacol. Sci..

[38] van der Esch M., Steultjens M., Harlaar J., Wolterbeek M., Knol J., Dekker J. (2008). Knee varus–valgus motion during gait—a measure of joint stability in patients with osteoarthritis?. Osteoarthr. Cartil..

[39] Bytyqi D., Shabani B., Lustig S., Cheze L., Karahoda Gjurgjeala N., Neyret P. (2014). Gait knee kinematic alterations in medial osteoarthritis: Three dimensional assessment. Int. Orthop..

[40] Shi J., Zhang K., Guo C., Yang Y., Xu Y., Wu J. (2024). A survey of label–noise deep learning for medical image analysis. Med. Image Anal..

[41] Li M., Zhu C. (2024). Noisy label processing for classification: A survey. arXiv.

[42] Pranto T.H., Noman A.A., Noor A., Deepty U.H., Rahman R.M. (2022). Effect of label noise on multi–class semantic segmentation: A case study on Bangladesh marine region. Appl. Artif. Intell..

[43] Fukuchi C.A., Fukuchi R.K., Duarte M. (2019). Effects of walking speed on gait biomechanics in healthy participants: A systematic review and meta-analysis. Syst. Rev..

[44] Hausdorff J.M. (2005). Gait variability: Methods, modeling and meaning. J. Neuroeng. Rehabil..

[45] Del Moral P., Nowaczyk S., Pashami S. (2022). Why is multiclass classification hard?. IEEE Access.

[46] Riaz M., Bashir M., Younas I. (2022). Metaheuristics based COVID–19 detection using medical images: A review. Comput. Biol. Med..

[47] Cao W., Mirjalili V., Raschka S. (2020). Rank consistent ordinal regression for neural networks with application to age estimation. Pattern Recognit. Lett..

[48] Shi X., Cao W., Raschka S. (2023). Deep neural networks for rank–consistent ordinal regression based on conditional probabilities. Pattern Anal. Appl..

[49] Deng J., Guo J., Xue N., Zafeiriou S. (2019). ArcFace: Additive angular margin loss for deep face recognition. Proceedings of the IEEE/CVF Conference on Computer Vision and Pattern Recognition.

[50] Cai X., Luo F., Qi W., Liu H. (2022). A semi-supervised object detection algorithm based on teacher-student models with strong-weak heads. Electronics.

[51] Singh D., Boubekki A., Jenssen R., Kampffmeyer M. (2025). SuperCM: Improving semi-supervised learning and domain adaptation through differentiable clustering. Pattern Recognit..

[52] Fu G., Zhang Z., Le W., Li J.B., Zhu Q.X., Niu F.Z., Chen H., Sun F.Y., Shen Y.H. (2023). A multi–scale pooling convolutional neural network for accurate steel surface defects classification. Front. Neurorobot..

[53] Zhang X., Nie Q., Xiao Z., Zhao J., Wu X., Guo P., Li R., Liu J., Wei Y.J., Pan Y. (2024). Dual-view pyramid pooling in deep neural networks for improved medical image classification and confidence calibration. arXiv.

[54] Furuta R., Sato Y. (2023). Seeking flat minima with mean teacher on semi- and weakly-supervised domain generalization for object detection. arXiv.

[55] Liu Q., Qi Y., Wang C. (2024). Multi-scale cross-layer fusion and center position network for pedestrian detection. J. King Saud Univ.-Comput. Inf. Sci..

[56] Tran D., Wang H., Torresani L., Ray J., LeCun Y., Paluri M. (2018). A closer look at spatiotemporal convolutions for action recognition. Proceedings of the IEEE Conference on Computer Vision and Pattern Recognition.

[57] Ghosh P., Hossain R.B., Zunaed M., Hasan T. (2025). Domain generalization for improved human activity recognition in office space videos using adaptive pre-processing. arXiv.

[58] Zhong Y., Zhou W.A., Wang Z. (2025). A survey of data augmentation in domain generalization. Neural Process. Lett..

